# Attentional modulation of inter-areal coherence explained by frequency shifts

**DOI:** 10.1016/j.neuroimage.2023.120256

**Published:** 2023-06-29

**Authors:** Jarrod Robert Dowdall, Marius Schneider, Martin Vinck

**Affiliations:** a Ernst Strüngmann Institute (ESI) for Neuroscience in Cooperation with Max Planck Society, Frankfurt am Main, Germany; b Robarts Research Institute, Western University, London, Ontario, Canada; c Donders Centre for Neuroscience, Department of Neurophysics, Radboud University, Nijmegen, the Netherlands

**Keywords:** Communication-through-coherence (CTC), Inter-areal coherence, Granger causality, Functional connectivity, Resonance, Source mixing

## Abstract

Inter-areal coherence has been hypothesized as a mechanism for inter-areal communication. Indeed, empirical studies have observed an increase in inter-areal coherence with attention. Yet, the mechanisms underlying changes in coherence remain largely unknown. Both attention and stimulus salience are associated with shifts in the peak frequency of gamma oscillations in V1, which suggests that the frequency of oscillations may play a role in facilitating changes in inter-areal communication and coherence. In this study, we used computational modeling to investigate how the peak frequency of a sender influences inter-areal coherence. We show that changes in the magnitude of coherence are largely determined by the peak frequency of the sender. However, the pattern of coherence depends on the intrinsic properties of the receiver, specifically whether the receiver integrates or resonates with its synaptic inputs. Because resonant receivers are frequency-selective, resonance has been proposed as a mechanism for selective communication. However, the pattern of coherence changes produced by a resonant receiver is inconsistent with empirical studies. By contrast, an integrator receiver does produce the pattern of coherence with frequency shifts in the sender observed in empirical studies. These results indicate that coherence can be a misleading measure of inter-areal interactions. This led us to develop a new measure of inter-areal interactions, which we refer to as Explained Power. We show that Explained Power maps directly to the signal transmitted by the sender filtered by the receiver, and thus provides a method to quantify the true signals transmitted between the sender and receiver. Together, these findings provide a model of changes in inter-areal coherence and Granger-causality as a result of frequency shifts.

## 1 Introduction

An outstanding question in neuroscience is how sparsely connected brain areas flexibly communicate behaviorally relevant information. It has been proposed that inter-areal synchronization flexibly gates neuronal communication according to behavioral and cognitive demands (Bressler and Kelso, 2001; Fries, 2005; Varela et al., 2001). Indeed, several studies have provided evidence for selective communication via coherence by showing that attention toward a stimulus leads to an increase in inter-areal coherence between V1 and V4 (Bosman et al., 2012; Ferro et al., 2021). Yet the mechanism by which coherence increases remains largely unknown. One key observation in these studies was there was that no difference in the oscillatory power between attention conditions, although there was a clear increase in the gamma peak frequency in V1 (Bosman et al., 2012; Ferro et al., 2021). Several studies have also shown that the peak frequency of gamma oscillations (30–100 Hz) in V1 systematically varies with low-level stimulus features often associated with bottom-up salience, such as color, contrast, and size (Das and Ray, 2018; Friedman-Hill et al., 2000; Gieselmann and Thiele, 2008; Gray and Prisco, 1997; Henrie and Shapley, 2005; Jia et al., 2013; Peter et al., 2019; Ray and Maunsell, 2010; Shirhatti and Ray, 2018). This suggests that shifts in the peak frequency of gamma oscillations may be a common mechanism by which both salient and attended stimuli are selectively processed and communicated between brain areas (Bosman et al., 2012; Fries, 2015).

Importantly, to understand how one sender can be more effective than another, it is necessary to understand how the receiver selects that sender’s synaptic inputs and not others (i.e., the receiver’s input transfer function). Broadly speaking, neurons can be classified as either integrators or resonators. Both integration and resonance have been observed experimentally and are emergent properties of biophysical models of neurons (Hodgkin and Huxley, 1952; Hutcheon and Yarom, 2000; Izhikevich, 2001). Whereas resonators amplify inputs that match the receiver’s resonant frequency, integrators are not frequency selective but rather tend to act as low-pass filters (Izhikevich, 2001). Due to the frequency selective properties of resonators, it has been proposed that the resonance may facilitate selective communication (“selective communication via resonance”) (Izhikevich et al., 2003). However, this appears at odds with the studies reporting a coherence increase with a greater frequency mismatch between V1 and V4 (Bosman et al., 2012; Ferro et al., 2021).

In this study, we addressed two fundamental aspects of inter-areal interactions: how to quantify the transmitted signal, and how the receiver responds to those inputs. Thus, we consider the general problem of how the oscillation frequency of the sender compared to the receiver results in selective communication according to the receiver’s input transfer function. Such a problem is not only relevant for understanding inter-areal neuronal communication but also more generally how neuronal populations selectively respond to certain frequencies and rhythms in their sensory input (e.g., speech, visual flicker) or brain stimulation (e.g., optogenetic, electric) (Adaikkan et al., 2019; Cardin et al., 2009; Doelling and Assaneo, 2021; Doelling et al., 2019; Duecker et al., 2021; Haegens and Golumbic, 2018; Jang et al., 2020; Lakatos et al., 2008; Lewis et al., 2021; Obleser and Kayser, 2019; Schroeder and Lakatos, 2009). In fact, modulating the frequency of stimuli, or stimulation, is a common method for probing the input-output function of neuronal populations (Cardin et al., 2009; Doelling and Assaneo, 2021; Doelling et al., 2019; Duecker et al., 2021; Lewis et al., 2021). Using theoretical analysis and numerical simulations, we systematically investigated the effect of frequency shifts in a source area on coherence with a downstream target area. Counter to the hypothesis of selective communication via resonance, we found that only the integrator receiver reproduced the experimentally observed changes in inter-areal coherence with frequency shifts in the sender (Bosman et al., 2012; Ferro et al., 2021). Furthermore, these results revealed that coherence is not a straightforward measure of inter-areal interactions. Thus, we were motivated to develop a new measure of inter-areal interactions, which we refer to as Explained Power. To that end, we show that Explained Power maps directly to the signal transmitted by the sender filtered by the receiver, providing an improved method to quantify frequency-specific signal transmission.

## 2 Methods

### 2.1 The source mixing model

We modeled the intrinsic activity of each area as a simple damped harmonic oscillator excited by stochastic drive implemented as an AR(2) model of the form

(1) $$x[t]=a_{1}x[t-1]+a_{2}x[t-2]+b_{1}\epsilon [t],$$

where *ϵ*[*t*] is the stochastic drive, such that *ϵ*[*t*] = *𝒩* (*μ*, *σ*^2^) where *μ* = 0, *σ*^2^ = 1, which drives the system producing oscillatory behavior as observed in the time-series *x*[*t*]. We chose to model the intrinsic activity in each area using pseudo-periodic AR(2) models because these models have been shown to reproduce the statistical properties of gamma oscillations in macaque primary visual cortex (V1) and provide mean-field approximations of E-I circuits driven by stochastic input (Spyropoulos et al., 2022).

In our model system, the time-series $x_{1}^{\left(int\right)}[t]$ is the intrinsic activity of a local network of neurons, and *x*_1_[*t*] is the observed time-series, which is a linear sum of the intrinsic activity, synaptic input from remote sources, and background fluctuations (e.g., 1/*f*). We first derive a simplified case of the Source Mixing in which there is one sender *x*_1_[*t*] and one receiver *x*_2_[*t*] with unidirectional (feedforward) connectivity in the absence of background fluctuations. In this simplified case the observed time-series are

(2) $$x_{1}[t]=x_{1}^{\left(int\right)}[t],$$

(3) $$x_{2}[t]=x_{2}^{\left(int\right)}[t]+wx_{1}^{\left(int\right)}[t-\tau ].$$

where *w* is the connectivity weight, which scales the output of *x*_1_ according the inter-areal connectivity strength between *x*_1_ and *x*_2_, and *τ* is the transmission delay.

Note, the Source Mixing model does not make an assumption about how the intrinsic activity in the sender and receiver is generated. Rather, the only assumption of the Source Mixing model is that the output of the sender is linearly superimposed on the intrinsic activity of the receiver. This means that the resulting coherence can be completely determined in terms of the shape of the observed power spectral densities.

The observed power spectral densities (PSD) of the sender *x*_1_ and receiver *x*_2_ are

(4) $$S_{11}(f)=S_{11}^{\left(int\right)}(f),$$

(5) $$S_{22}(f)=S_{22}^{\left(int\right)}(f)+w^{2}S_{11}^{\left(int\right)}(f).$$

The stochastic drive in the sender *ϵ*_1_[*t*] and receiver *ϵ*_2_[*t* + *τ*] were uncorrelated for all *τ*. Thus the observed cross-spectral density (CSD) between the sender and receiver reduces to the output of *x*_1_ scaled by the connectivity weight

(6) $$S_{12}(f)=wS_{11}^{\left(int\right)}(f).$$

Therefore, coherence given Source Mixing is

(7) $$\begin{array}{l} C_{12}^{2}(f)\approx \frac{{\left|S_{12}(f)\right|}^{2}}{S_{11}(f)S_{22}(f)}\\ \;\;\;\;\;\;\;\;\;\;\;\;\;\;\;\approx \frac{w^{2}{\left(S_{11}^{\left(int\right)}(f)\right)}^{2}}{S_{11}^{\left(int\right)}(f)\left(S_{22}^{\left(int\right)}(f)+w^{2}S_{11}^{\left(int\right)}(f)\right)}\\ \;\;\;\;\;\;\;\;\;\;\;\;\;\;\approx \frac{w^{2}S_{11}^{\left(int\right)}(f)}{S_{22}^{\left(int\right)}(f)+w^{2}S_{11}^{\left(int\right)}(f)}. \end{array}$$

Let *α*(*f*) be the power ratio between the sender and receiver

(8) $$\alpha (f)\equiv \frac{S_{11}^{\left(int\right)}(f)}{S_{22}^{\left(int\right)}(f)}.$$

Substituting *α*(*f*) into Eq. (7)

(9) $$C_{12}^{2}(f)=\frac{w^{2}\alpha (f)}{w^{2}\alpha (f)+1}.$$

Let *z* = ln *w*^2^*α*(*f*), where *w*^2^*α*(*f*) > 0, shows coherence follows the standard logistic function

(10) $$C_{12}^{2}(f)=\frac{e^{z}}{e^{z}+1}=\frac{1}{1+e^{-z}}.$$

### 2.2 Source mixing with an input transfer function

In the Source Mixing model with an input transfer function, the sender’s output *x*_1_ is filtered by the receiver’s input transfer function *h*_*input*_ as follows:

(11) $$x_{1}[t]=x_{1}^{\left(int\right)}[t],$$

(12) $$x_{2}[t]=x_{2}^{\left(int\right)}[t]+w\left(h_{input\;}*x_{1}^{\left(int\right)}\right)[t-\tau ],$$

where * denotes convolution.

The PSD of the sender *x*_1_ and receiver *x*_2_ is then

(13) $$S_{11}(f)=S_{11}^{\left(int\right)}(f),$$

(14) $$S_{22}(f)=S_{22}^{\left(int\right)}(f)+w^{2}S_{11}^{\left(int\right)}(f)H_{input}^{2}(f).$$

As before, *ϵ*_1_[*t*] and *ϵ*_2_[*t*] were uncorrelated, thus the cross-spectral density (CSD) simplifies to

(15) $$S_{12}(f)=wS_{11}^{\left(int\right)}(f)H_{input}(f).$$

Thus coherence is defined as

(16) $$\begin{array}{l} C_{12}^{2}(f)=\frac{w^{2}{\left(S_{11}^{\left(int\right)}(f)\right)}^{2}H_{input\;}^{2}(f)}{S_{11}^{(int)\;}(f)\left(S_{22}^{(int)\;}(f)+w^{2}S_{11}^{\left(int\right)}(f)H_{input\;}^{2}(f)\right)}\\ \;\;\;\;\;\;\;\;\;\;\;\;\;\;\;\;=\frac{w^{2}\alpha (f)H_{input\;}^{2}(f)}{w^{2}\alpha (f)H_{input\;}^{2}(f)+1}. \end{array}$$

Therefore, without loss of generality, a linear transform applied to the receiver’s input follows the simplified Source Mixing model Eq. (9), and coherence maps non-linearly to the sender-receiver power ratio *α* scaled by the connectivity weight *w* and receiver’s input transfer function *H*_*input*_.

### 2.3 Source mixing with matching transfer functions

An interesting property emerges when the receiver’s input transfer function *H*_*input*_ matches the receiver’s power spectrum

(17) $$S_{22}^{\left(int\right)}(f)={\left|S_{\epsilon_{2}}(f)H_{input}(f)\right|}^{2},$$

where ${\left|S_{\epsilon_{2}}(f)\right|}^{2}$ is the power spectrum of the stochastic drive *ϵ*_2_.

Note, the power spectrum of a time-series is the stochastic drive *ϵ*_2_ is described by its time-domain variance $\sigma_{\epsilon_{2}}^{2}$

(18) $$E\left[{\left|S_{\epsilon_{2}}(f)\right|}^{2}\right]=\sigma_{\epsilon_{2}}^{2}.$$

Thus, Eq. (16) simplifies to

(19) $$C_{12}^{2}(f)=\frac{w^{2}S_{11}^{\left(int\right)}(f)}{w^{2}S_{11}^{\left(int\right)}(f)+\sigma_{\epsilon_{2}}^{2}}.$$

Therefore, in the special case where the receiver’s input transfer function *H*_*input*_ matches its power spectrum, coherence is invariant to the receiver’s power spectrum. Note, although coherence still depends on the receiver’s variance $\sigma_{\epsilon_{2}}^{2}$, the frequency dependence has been removed and coherence no longer depends on the power ratio between the sender and receiver per frequency. However, this result implies coherence only becomes independent of the receiver when the power in the receiver is perfectly compensated for by the receiver’s input transfer function. Any uncompensated power in the receiver, such as background 1/*f* and any linear mixing from additional inputs.

### 2.4 Explained power

We have shown that coherence can be a misleading measure of inter-areal interactions. For instance, coherence differences are misleading when there are frequency-dependent interactions between the sender(s) and the receiver. Here we motivate a more veridical measure of inter-areal interactions, which we refer to as Explained Power.

Note that magnitude-squared coherence is akin to the coefficient of determination in linear regression analysis (i.e., explained variance). Therefore, Explained Power *E*_12_ can be computed by taking the product of magnitude squared coherence $C_{12}^{2}$ and the receiver’s power spectrum *S*_22_

(20) $$\begin{array}{c} E_{12}(f)\equiv S_{22}(f)C_{12}^{2}(f)\\ \;\;\;\;\;\;\;\equiv \frac{{\left|S_{12}(f)\right|}^{2}}{S_{11}(f)}. \end{array}$$

Expanding the definition above shows that explained power captures the sender’s projected signal scaled by the connectivity weight *w* and receiver’s input transfer function *H*_*input*_,

(21) $$\begin{array}{c} E_{12}(f)=S_{22}(f)\frac{w^{2}{\left|S_{11}(f)\right|}^{2}H_{input\;}^{2}}{S_{11}(f)S_{22}(f)}\\ =w^{2}S_{11}(f)H_{input\;}^{2}. \end{array}$$

In this example, the interaction between the sender and receiver is uni-directional, which leads to a straightforward interpretation of Explained Power.

We have assumed the observed power spectrum of the sender is equivalent to the projected signal from the sender. In practice, this may not be the case, and Explained Power will depend on the spectrum of the unprojected part of the sender, such as additive background fluctuations (e.g., 1/*f*). This naive estimation of Explained Power will tend to underestimate the signal transmitted by the sender. However, it is possible to correct this underestimation in Explained Power as follows

(22) $${\hat{E}}_{12}(f)\equiv \frac{{\left|S_{12}(f)\right|}^{2}}{\left(S_{11}(f)-S_{baseline}(f)\right)},$$

provided estimating the unprojected signal can be reasonably motivated (e.g., with a pre-stimulus or baseline condition). Note that the derivation here is just based on the expression of the coherence magnitude squared.

Note, this correction comes at the expense of the potential overestimation, i.e., can lead to negative values, and is therefore, unreliable as *S*_11_ (*f*) − *S*_*baseline*_ (*f*) *→* 0. Taking these limitations into consideration, ${\hat{E}}_{12}$ should only be estimated over a frequency range where the power is maximal in the sender (e.g., full width at half-maximum).

We note that the equivalence of the integral of the power spectral density and time-domain variance of *x*_2_

(23) $$Var\left[x_{2}\right]=\int S_{22}(f)df.$$

which leads to a unitless measure of Explained Power we refer to as the Proportion of Explained Power (PEP).

PEP (see Fig. 5(g)–(i)) is then defined as

(24) $${\tilde{E}}_{12}\equiv \frac{E_{12}}{\int S_{22}(f)df}.$$

Furthermore, it follows then that the integral of explained power is equivalent to the total explained variance

(25) $$Var_{2,exp}=\int S_{22}(f)C_{12}^{2}(f)df.$$

The Explained Power measure is further supported by the relationship of coherence with the Wiener filter (Wiener, 1949). Wiener (1949) showed that for an acausal filter, where a signal *x*_2_ is predicted from a linear combination of *x*_1_ (at different delays), the minimum mean squared error (in the prediction of *x*_2_) is given by

(26) $$Var_{2,\text{residual }}=\int S_{22}\left(1-C_{12}^{2}(f)\right))df,$$

and thus,

(27) $$\begin{array}{l} \int S_{22}C_{12}^{2}(f)=Var[x_{2}]-Var_{2,\text{residual }}\\ \int E_{12}(f)df=Var[x_{2}]-Var_{2,\text{residual}}, \end{array}$$

where *V ar*_2,residual_ is the residual (or unexplained) variance, *Var* [*x*_2_] is the variance of *x*_2_, and $C_{12}^{2}$ is magnitude squared coherence between *x*_1_ and *x*_2_. Note that normalizing the residual variance by the total variance is equivalent to 1 − *∫* PEP(*f*) *df*

(28) $$\int {\tilde{E}}_{12}\;df=1-\frac{Var_{2,\text{residual }}}{Var[x_{2}]}.$$

Note, when the interaction between the sender and receiver is bidirectional, separating feedforward and feedback Explained Power is not entirely straightforward. It would appear reasonable to isolate feedforward and feedback Explained Power utilizing the causal power equations derived from Granger–Geweke causality (GGC) (Dhamala et al., 2018). However, this approach inherits the biases known to affect GGC, namely the issue that additive noise in the sender can reverse the direction of causality (Vinck et al., 2015). A more problematic issue is that bidirectional phase delays lead to interference patterns in the coherence spectra that mask the true underlying feedforward and feedback causal interactions. A full discussion on this topic is beyond the scope of this manuscript, but we have briefly noted this issue here as it generally pertains to causal inference based on phase (time) delays applied to bidirectional systems.

### 2.5 Determining the coefficients of the autoregressive model

For our simulations, we modeled the intrinsic activity of each cortical ensemble with a causal second-order autoregressive model with complex roots. The impulse response function (*h*) of an AR(2) model with complex roots is a dampened oscillation. When driven by white noise, an AR(2) model can exhibit pseudo-periodic stochastic fluctuations around its resonant frequency.

An autoregressive process of order *m* is defined as

(29) $$y_{t}=a_{0}+\sum_{k=1}^{m}a_{k}y_{t-k}+\epsilon_{t},$$

where *ϵ_t_* is a white noise process *ϵ_t_* ~ *𝒩* (*μ*, *σ*^2^) with mean *μ* = 0, variance *σ*^2^, and expected value 𝔼 [*y*_*t*_ ] = *a*_0_.

We were particularly interested in AR(2) models with roots *λ*_1,2_ that form a complex conjugate pair

(30) $$\lambda_{1,2}=\frac{a_{1}\pm i\sqrt{-\left(a_{1}^{2}+4a_{2}\right)}}{2}.$$

The concentration of spectral energy, or peakiness, of an AR(2) with complex roots is proportional to the modulus, *R*, of its roots *λ*_1,2_, where 0 < *R* < 1. When *R* is close to 1 the resulting time-series is almost perfectly sinusoidal, and as *R* approaches zero, the power spectrum of the time-series becomes less peaked (i.e., flat).

*R* is determined by taking the Pythagorean sum of the real and imaginary parts of the complex roots (Eq. (30))

(31) $$\begin{array}{l} R^{2}=\frac{a_{1}^{2}}{4}+\frac{-\left(a_{1}^{2}+4a_{2}\right)}{4}\\ \;\;\;\;\;\;\;=-a_{2}. \end{array}$$

Note, the boundary conditions $a_{2}=-0.25a_{1}^{2}$ and *a*_2_ = −1 force −1 < *a*_2_ < 0, which ensures *R* is real-valued.

The analytic expression of the one-sided power spectrum of an AR(2) process is

(32) $$S_{yy}(\omega )=\frac{\sigma_{\epsilon }^{2}}{{\left|1-a_{1}e^{-i\omega }-a_{2}e^{-i2\omega }\right|}^{2}},$$

where *ω* is the normalized frequency in radians per sample (i.e., $\omega =2\pi \frac{f}{fs}$, for frequency *f* and sampling rate *fs*).

For simplicity, Eq. (32) can be written in the real-domain as

(33) $$S_{yy}(\omega )=\frac{\sigma_{\epsilon }^{2}}{1+a_{1}^{2}+a_{2}^{2}-2a_{1}\left(1-a_{2}\right)\text{cos}(\omega )-2a_{2}\text{cos}(2\omega )}.$$

To derive the analytical expression for the AR(2) coefficients as a function of the peak frequency, *ω*_max_, we only need to take the partial derivative of Eq. (33) over *ω*. That is, the power spectrum of an AR(2) reaches its peak when the denominator in Eq. (33) reaches its minimum. The analytic expression for the peak frequency *ω*_max_ is then

(34) $$\omega_{max\;}={\text{cos}}^{-1}\left(\frac{a_{1}\left(a_{2}-1\right)}{4a_{2}}\right),$$

where *ω*_max_ is the peak frequency in radians per sample.

Thus, Eqs. (31) and (34) allow us to derive the coefficients of an AR(2) with complex roots with the desired modulus *R* and peak frequency *ω*_max_

(35) $$a_{1}=\frac{4a_{2}\text{cos}\left(\omega_{max}\right)}{a_{2}-1},$$

(36) $$a_{2}=-R^{2}.$$

Notice that Eq. (35) implies *a*_2_ < *a*_1_ /(*a*_1_ ± 4).

Thus, the AR(2) models of interest in this study were those enclosed by the boundary conditions

(37) $$0<a_{1}<2,$$

(38) $$-1<a_{2}<\frac{a_{1}}{a_{1}-4}.$$

#### 2.5.1 Controlling peak power while varying other parameters

In our simulations, we wanted to control the maximum power (peak power) at the resonant frequency *ω*_max_, while changing other parameters such as the modulus of the eigenvalue *R*, or the variance of the time-series. To that end, we derived the equations that describe the peak power and variance as a function of *R*.

The equation for peak power is easily derived by substituting Eq. (35) into Eq. (33) and solving for *S*_*yy*_ (*ω*_max_) = *S*_max_

(39) $$S_{max}=\frac{\sigma_{\epsilon }^{2}{\left(a_{2}-1\right)}^{2}}{{\left(a_{2}+1\right)}^{2}\left(a_{2}^{2}+2a_{2}\text{cos}\left(2\omega_{max}\right)+1\right)}.$$

Note, this equation can be further written in terms of *R* by substituting in Eq. (36).

In order to control the time-domain variance (i.e., integral of the PSD) of the AR(2) model, we first derived the equation for the variance of an AR(2) process in terms of its coefficients

(40) $$Var\left[y_{t}\right]=\frac{\left(1-a_{2}\right)\sigma_{\epsilon }^{2}}{\left(1+a_{2}\right)\left({\left(1-a_{2}\right)}^{2}-a_{1}^{2}\right)}.$$

Eq. (35) was then substituted in to remove *a*_1_

(41) $$Var\left[y_{t}\right]=\left(\frac{-{\left(a_{2}-1\right)}^{3}}{a_{2}+1}\right)\left(\frac{\sigma_{\epsilon }^{2}}{{\left(a_{2}-1\right)}^{4}-16a_{2}^{2}\;{\text{cos}}^{2}\left(\omega_{max}\right)}\right).$$

Finally, Eqs. (39) and (36) can then be substituted into Eq. (41),

(42) $$Var\left[y_{t}\right]=-S_{max}\frac{\left(R^{4}-1\right)\left(R^{4}-2R^{2}\text{cos}\left(2\omega_{max}\right)+1\right)}{{\left(R^{2}+1\right)}^{4}-16R^{4}{\text{cos}}^{2}\left(\omega_{max}\right)}.$$

Notice that *V ar* [*y*_*t*_] ∈ [0, *S*_max_], thus as *V ar* [*y*_*t*_] *→* 0 the waveform tends to a near perfect sinusoidal oscillation, whereas when *V ar* [*y*_*t*_] *→ S*_max_ the power spectrum flattens.

We used the Matlab function vpasolve() to solve Eqs. (39) and (42) to determine *R* given the desired *ω*_max_, *S*_max_, or *V ar* [*y*_*t*_].

#### 2.5.2 Resonator input transfer function

As defined previously, we modeled the resonator input transfer function with a second-order autoregressive model. The resonator’s input transfer function was always identical to the receiver’s intrinsic power spectrum (peak frequency at 60 Hz). However, because we set the peak power of the intrinsic oscillations equal to 1, this would have meant the input transfer function would act as a unit-gain (at the resonant frequency) band-pass filter. Therefore, we added a gain factor *g* that converted the input transfer function from unit gain to amplifying at the resonant frequency when *g* > 1. Note that the gain factor did not change the pattern of coherence across frequencies, only the absolute magnitude.

Thus, the resonator input transfer function was

(43) $$H_{input\;}=g\;H_{2},$$

where *H*_2_ is the receiver’s intrinsic transfer function. The gain factor for the bivariate sender-receiver simulations with a resonator was *g* = 1.5 (Figs. 2 and 5). For the triplet model simulation, the resonator gain factor was *g* = 1.6 (Fig. 3(d) and (e)). Note, the gain factor only applied to the resonator transfer function, and was otherwise equal to 1.

#### 2.5.3 Integrator input transfer function

We modeled the integrator input transfer function with an exponential decay

(44) $$x[n]=(1-\alpha )x[n-1]+\alpha x^{\left(proj\right)}[n],$$

where *x*^(*proj*)^ is the signal projected to, and filtered by the receiver’s input transfer function, and *α* is

(45) $$\alpha =1-e^{-\frac{\text{Δ}T}{\tau }}.$$

The transfer function of the exponential filter is

(46) $$H(\omega )=\frac{\alpha }{1-(1-\alpha )e^{-j\omega }}.$$

The exponential filter is a low-pass filter, and thus it is possible to describe the filter by its corner frequency (i.e., the frequency where the power of the input is attenuated by 3 decibels).

From the spectrum, we derived the equation for the corner frequency

(47) $$f_{corner\;}=\frac{fs}{2\pi }{\text{cos}}^{-1}\left(1-\frac{\alpha^{2}}{2(1-\alpha )}\right),$$

where *fs* = 1000 Hz is the sampling frequency of the time-series.

### 2.6 *Background* 1/*f fluctuations and numerical simulations*

Note that in the presence of 1/*f* fluctuations, we can describe

(48) $$x^{\left(obs\right)}[t]=x^{\left(int\right)}[t]+\eta_{x}[t],$$

where *η*_*x*_ [*t*] is a background term containing 1/*f* fluctuations. We assumed that these 1/*f* fluctuations were not projected, nor correlated with each area’s intrinsic activity.

The power spectrum of 1/*f* is approximately equal to the inverse of the frequency

(49) $$S(f)\propto \frac{1}{f}.$$

We simulated the 1/*f* spectrum according to

(50) $$S(f)=\frac{f_{0}}{f}P,$$

then inverse Fourier transformed the spectrum to arrive at the time-domain coefficients of an *N*^*th*^ order FIR filter, where N was equal to the number of samples in each simulated time-series epoch. For all simulations that included background 1/*f*, *f*_0_ was set to the receiver’s peak frequency (60 Hz) and *P* = 3^−1^.

The peak power for all intrinsic oscillations (simulated with AR(2) filters) was always equal to 1.

The sampling rate for all simulations was *fs* = 1000. The epoch length was *N* = 1000 samples, and 2500 independent epochs were generated for each run of a simulation. Each simulation was run 15 times (with a unique seed for the random number generator), the first 50,000 samples were discarded, and the results overall runs were averaged.

Note, the pattern of coherence changes was not significantly altered by changing the parameters of the simulations. That is, the result that coherence increased with increasing sender-receiver frequency mismatch for the integrator receiver, and decreased for the resonator receiver held true over a wide range of the parameter space.

## 3 Results

Our primary question was how shifts in the oscillation frequency of the sender lead to changes in coherence between sender and receiver. To investigate this, we simulated the local field potential (LFP) of two anatomically connected brain areas with pseudo-periodic causal autoregressive (AR) models. Pseudo-periodic AR models reproduce the statistical properties of stationary gamma oscillations in V1 and provide mean-field approximations of E-I circuits driven by stochastic input (Spyropoulos et al., 2022). However, note that the results presented here do not depend on AR models specifically. To elucidate how the sender oscillation frequency affects coherence, we systematically varied the sender frequency and computed coherence between the sender and receiver.

Recently, Schneider et al. (2021) demonstrated that differences in the magnitude of inter-areal coherence can be explained by differences in the oscillatory power of the source projecting areas. The authors derived an analytical model of coherence, referred to as the Source Mixing model, which can be expressed in terms of the input-output relationships of linear systems perturbed by stochastic noise (Bendat and Piersol, 2010; Trueblood and Alspach, 1977). Conceptually, the Source Mixing model follows from the non-local nature of the LFP and relies on the assumption that input spikes from the sender depolarize target cells in the receiver leading to correlated fluctuations in the LFPs of the sender and receiver.

However, the Source Mixing model presented in Schneider et al. (2021) assumed the receiver responds to all inputs identically independent of their frequency (i.e., the receiver acts as an all-pass filter). Yet, it is known that some neurons integrate their synaptic inputs (i.e., act as a low-pass filter), and other neurons resonate at a particular frequency according to their synaptic inputs (i.e., act as a band-pass filter) (Hutcheon and Yarom, 2000; Izhikevich et al., 2003). Importantly, integration and resonance represent distinct forms of excitability of neurons according to the temporal structure of their synaptic input. We reasoned that coherence should depend on the intrinsic filtering properties of neurons.

To explore the filtering effect of the receiver on coherence we compared the effect of frequency shifts in the sender with an integrator and a resonator receiver. Although integration and resonance are conceptually distinct and have opposing effects on coherence with frequency shifts in the sender, both are reducible to the same analytical equation (see Section 2.2).

### 3.1 Integrator vs. resonator: feedforward communication between sender-receiver pairs

**Fig. 1:**
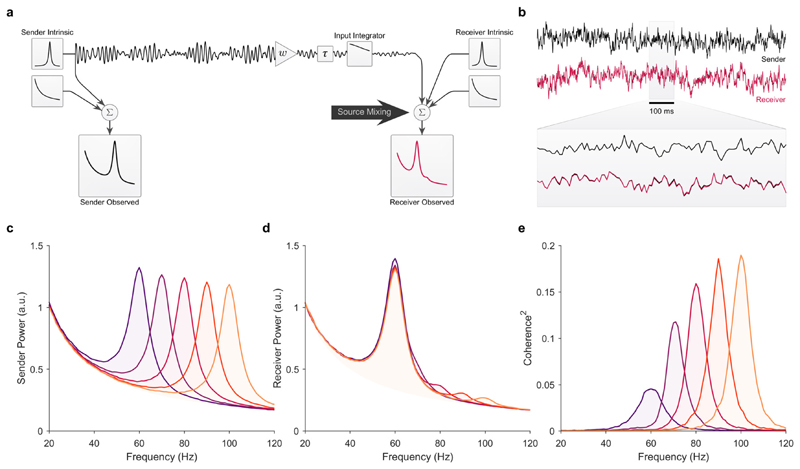
Source Mixing with an integrating receiver. Comparison between five sender-receiver pairs with unidirectional feedforward communication in the presence of additive 1/*f* background fluctuations. (a) Simulation circuit diagram. For these simulations, the feedforward connectivity weight was *w* = 0.35 and the inter-areal delay was *τ* = 3 ms. The receiver’s input transfer function was a low-pass filter (exponential moving average, cutoff: 100 Hz) applied identically to each sender’s output. Note, Only the oscillatory part of the sender was projected. The max power (peak in the PSD) of the oscillatory (i.e., transmitted) signals in all senders was identical, that is, only the peak frequency was varied. (b) Example of 1 s of the simulated time-series of the sender (black line) and receiver (red line). For the receiver’s time-series example only, the receiver’s observed time series without (black line) the projected signal from the sender is plotted under the time-series with (red line) the projected signal from the sender. This illustrates the contribution of the sender’s projected signal relative to the intrinsic (and 1/*f*) fluctuations in the receiver. (c) The observed PSDs of the five senders with a peak frequency at 60, 70, 80, 90, and 100 Hz, respectively. Note, the observed sender PSD is the sum of the PSDs of the sender’s intrinsic oscillatory signal and background 1/*f*. (d) The observed PSDs of the receiver paired with each of the five senders. The receiver’s PSD was the same in all sender-receiver pairs, i.e., only the sender’s frequency was shifted. Note, the observed receiver PSD is the sum of the PSDs of the receiver’s intrinsic signal, the signal received from the sender, and background 1/*f*. (e) Magnitude squared coherence between the observed power spectra for each sender-receiver pair. Note, the intrinsic oscillations, and background 1/*f*, in the sender and receiver, were fully uncorrelated. Thus, all coherence was due to the signal projected from the sender to the receiver. (For interpretation of the references to color in this figure legend, the reader is referred to the web version of this article.)

We first simulated a set of bivariate models with unidirectional feedforward communication between a single sender and receiver. We simulated five sender-receiver pairs in which the sender’s peak frequency was varied between 60 and 100 Hz while the receiver’s intrinsic power spectrum remained constant (peak frequency at 60 Hz). The output of the sender was scaled by the connectivity weight (*w* = 0.35), passed through the receiver’s input transfer function (*H*_*input*_), delayed (*τ* = 3 ms), and then superimposed on the intrinsic activity of the receiver (Fig. 1(a)). Note, only the oscillatory part of the sender was projected to the receiver (see Section 2.1). Furthermore, we simplified our simulations by assuming the transmitted signal from the sender is perfectly correlated with the oscillatory part of the LFP. That is, we explicitly avoided introducing any frequency-dependent transforms between the transmitted signal and LFP in the sender in order to isolate the effect of the receiver’s power spectrum and input transfer function on coherence. Thus, the intrinsic oscillations in the sender and receiver can be assumed to directly reflect the average instantaneous firing rate within each area. We refer to the superposition of an area’s intrinsic activity, additive background 1/*f* fluctuations, and any inputs to that area as the observed activity in that area. We always computed coherence between observed signals in the sender and receiver.

In Fig. 1, we show the results for an integrator receiver modeled as a first-order exponential moving average (cutoff: 100 Hz, see Section 2.5.3). We observed that coherence increased as the sender frequency shifted away from the peak frequency of the receiver (Fig. 1(e)), which was also true for simulations with and without additive 1/*f* fluctuations. The effect of sender frequency on coherence with an integrator receiver was qualitatively similar given a flat (all-pass) input transfer function (see Fig. 5(a) and (b)). The increase in coherence is explained by the change in the power ratio between the sender and receiver as the sender’s peak frequency shifts away from the peak of the power in the receiver. That is, as the sender’s peak frequency shifted further away from the peak power in the receiver, the sender’s projected signal was less diluted by the power in the receiver and coherence increased.

**Fig. 2:**
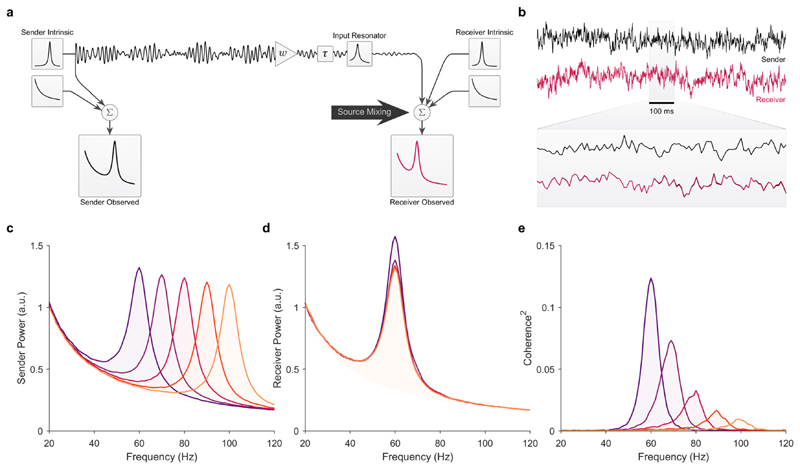
Source Mixing with a resonant receiver. Comparison between five sender-receiver pairs with unidirectional feedforward communication in the presence of additive 1/*f* background fluctuations. (a) Simulation circuit diagram. For these simulations, the feedforward connectivity weight was *w* = 0.35 and the inter-areal delay was *τ* = 3 ms. The receiver’s input transfer function was a band-pass filter (AR(2) model with a peak at 60 Hz) applied identically to each sender’s output. Note, only the oscillatory part of the sender was projected. (b) Example of 1 s of the simulated time-series of the sender (black line) and receiver (red line). For the receiver’s time-series example only, the receiver’s observed time-series without (black line) the projected signal from the sender is plotted under the time-series with (red line) the projected signal from the sender. This illustrates the contribution of the sender’s projected signal relative to the intrinsic (and 1/*f*) fluctuations in the receiver. Note, the intrinsic activity in these simulations is identical to the simulations shown in Fig. 1(b). The only difference between these simulations was the receiver’s input transfer function, yet the effect on coherence was the complete opposite. (c) The observed PSDs of the five senders with a peak frequency at 60, 70, 80, 90, and 100 Hz, respectively. (d) The observed PSDs of the receiver paired with each of the five senders. The receiver’s PSD was the same in all sender-receiver pairs, i.e., only the sender’s frequency was shifted. (e) Magnitude squared coherence between the observed power spectra for each sender-receiver pair. Note, the intrinsic oscillations in the sender and receiver were fully uncorrelated. Thus, all coherence was due to the signal projected from the sender to the receiver. (For interpretation of the references to color in this figure legend, the reader is referred to the web version of this article.)

In Fig. 2, we show the results for a resonant receiver modeled as an AR(2) process (peak frequency at 60 Hz, see Section 2.5.2). We assumed that the receiver’s input transfer function was identical to the intrinsic power spectrum of the receiver. Contrary to the integrator receiver, coherence decreased with a resonant receiver as the sender frequency shifted away from the receiver’s peak frequency (Fig. 2(e)). Because the resonant receiver amplifies inputs at its resonant frequency and suppresses frequencies further away from its resonant frequency, it directly influences the power ratio of the sender’s projected signal relative to the receiver’s intrinsic power. The net effect is that the resonant receiver dampens the rise in coherence with increasing sender-receiver frequency mismatch, unlike the integrator receiver. In general, this effect applies to all resonators, however, a special case arises when the receiver’s input transfer function matches its intrinsic power spectrum, as in our simulation. In this special case, the receiver’s input transfer function perfectly compensates for the intrinsic power in the receiver, and the magnitude of coherence becomes invariant to the intrinsic power spectrum of the receiver. The change in coherence with increasing sender frequency shown in Fig. 2(e) arises because of the presence of additive background fluctuations in the receiver. Thus, in the absence of background 1/*f* the magnitude of coherence does not change with shifts in the sender frequency (see Fig. 5(c)). Note, we chose to match the input transfer function of the receiver to its power spectrum, because this was the most parsimonious case.

### 3.2 The triplet model: feedforward competition between two senders

Next, we aimed to simulate the study design in empirical studies that observed inter-areal coherence differences between two competing senders with different peak frequencies, but equal peak power, projecting to the same receiver (Bosman et al., 2012; Ferro et al., 2021). We refer to this simulation as the triplet model.

**Fig. 3:**
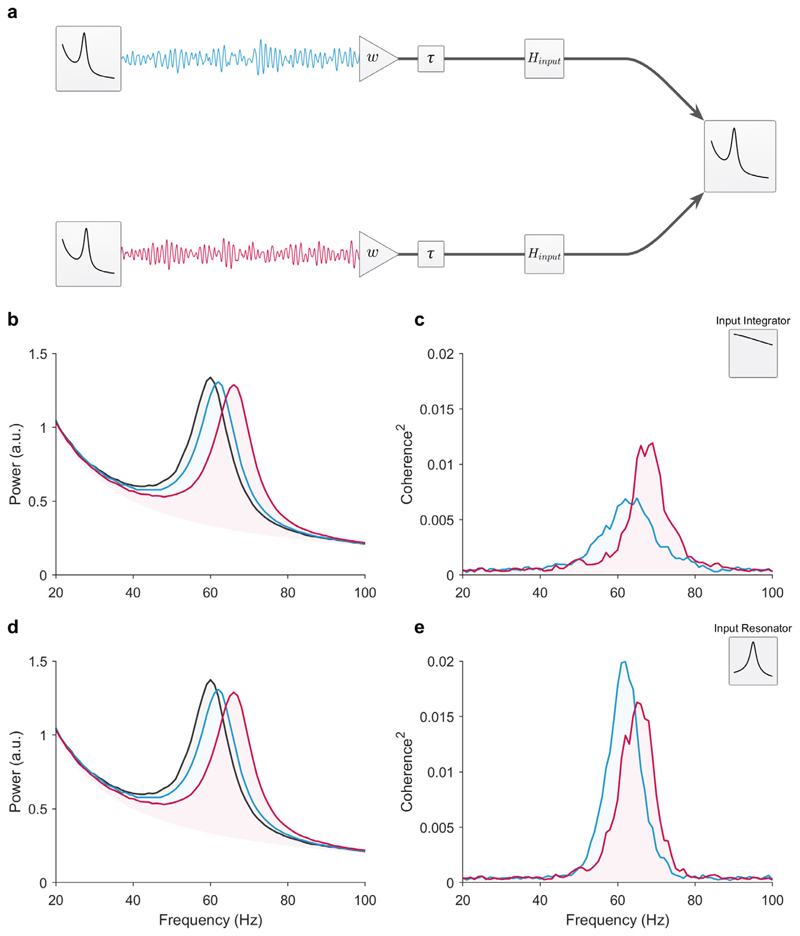
The triplet: Feedforward competition between two senders with different frequencies. (a) Illustration of the circuit. For these simulations, the feedforward connectivity weight was *w*_*ff*_ = 0.12 and the inter-areal delay was *τ* = 3 ms. The activity from both senders was filtered by the receiver’s input transfer function, *H*_*input*_, and superimposed on the receiver’s intrinsic activity. Note, only the oscillatory part of the sender was projected from each sender. (b-c) Simulation with an integrator receiver modeled as an exponential moving average low-pass filter (cutoff: 100 Hz). (b) Observed power spectra of the senders with a peak frequency of 62 and 66 Hz (blue and red lines, respectively) and the receiver with a peak at 60 Hz (black line). (c) Coherence between the receiver with each sender. (d-e) Same as the integrator receiver, but now for the resonant receiver modeled with an AR(2) model (peak frequency at 60 Hz). All intrinsic oscillations were uncorrelated to each other. Thus, all coherence was due to the signals projected from the sender to the receiver. (For interpretation of the references to color in this figure legend, the reader is referred to the web version of this article.)

First, we ran the triplet simulation with unidirectional feedforward connectivity (*w*_*ff*_ = 0.12, *τ* = 3 ms) from the senders (peak frequencies at 62 and 66 Hz, respectively) to the receiver (peak frequency at 60 Hz). This simulation was otherwise identical to the previous simulations with bivariate sender-receiver pairs. Similarly, we compared the influence of the receiver’s transfer function (the integrator and resonator, as previously defined) on coherence in the presence of 1/*f* background fluctuations (Fig. 3(c)–(e)). Note, the senders were unconnected and uncorrelated with each other.

Consistent with the bivariate simulations, the magnitude of the coherence was greater for the higher frequency sender with an integrator receiver (Fig. 3(c)), and lower with the resonant receiver (Fig. 3(e)). Together these results indicate that source mixing with frequency differences between two senders, feedforward connectivity, and an integrator receiver is sufficient to explain the empirically observed increase in V1–V4 coherence with attention (Bosman et al., 2012; Ferro et al., 2021). However, thus far our simulations have ignored the known anatomical feedback connectivity from V4 to V1 (Markov et al., 2014a; 2014b).

**Fig. 4:**
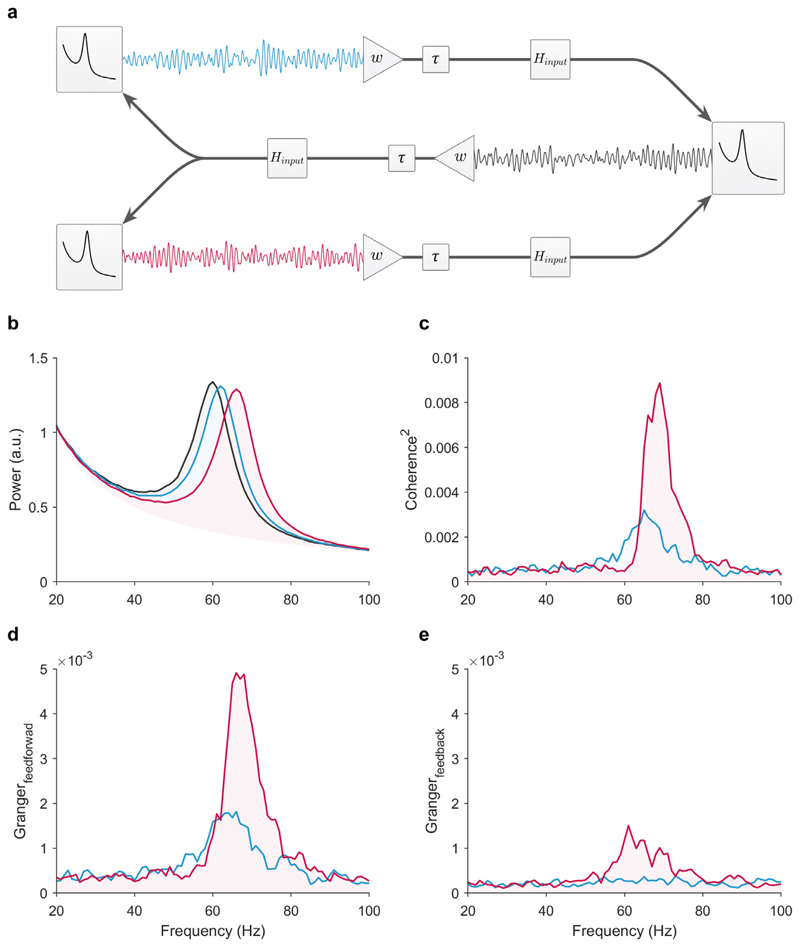
The triplet: feedforward competition with feedback. (a) Illustration of the circuit. For these simulations, the feedforward connec-tivity weight was *w*_*ff*_ = 0.12, and the feedback connectivity was *w*_*fb*_ = 0.06. The interareal delay was *τ* = 3 ms for both feedforward and feedback communication. Both senders’ received identical feedback from the receiver. Note, only the oscillatory part of the sender was projected from each sender. Here we only show the results for the simulation with an integrator receiver modeled as an exponential moving average low-pass filter (cutoff: 100 Hz). (b) Observed power spectra of the senders with a peak frequency of 62 and 66 Hz (blue and red lines, respectively) and the receiver with a peak at 60 Hz (black line). (c) Coherence between the receiver with each sender. Note, the coherence pattern shows a strong increase similar to (Bosman et al., 2012). (d) Feedforward non-parametric Granger–Geweke Causality between the receiver with each sender. (e) Feedback non-parametric Granger–Geweke Causality. (For interpretation of the references to color in this figure legend, the reader is referred to the web version of this article.)

We then asked whether the pattern of coherence observed with the integrator receiver would change with feedback (see Fig. 4). To that end, we added feedback (*w*_*fb*_ = 0.06, *τ* = 3 ms) to the triplet model, but otherwise, the model remained identical to the feedforward-only model. Note, the receiver projected the identical signal to both senders with the same connectivity weight and delay. In this simulation, both the senders and receiver were modeled as integrators with the identical input transfer function as defined previously (exponential moving average, cutoff: 100 Hz). In addition to computing coherence, for this simulation only, we also computed non-parametric Granger–Geweke Causality (Dhamala et al., 2018) (Fig. 4(d) and (e)).

The pattern of coherence observed in the feedback triplet model with integrator transfer functions was consistent with the results from the feedforward-only model. However, with feedback, the difference in coherence between the two senders and the receiver was magnified. A complete explanation as to why the coherence difference is magnified with feedback is beyond the scope of this manuscript (Dowdall and Vinck, 2023). However, we have noted that this is an artifact of the way coherence is computed. That is, this is a methodological issue, and further points to the difficulty in interpreting coherence differences as reflecting true differences in the strength of inter-areal communication.

### 3.3 Explained power

**Fig. 5:**
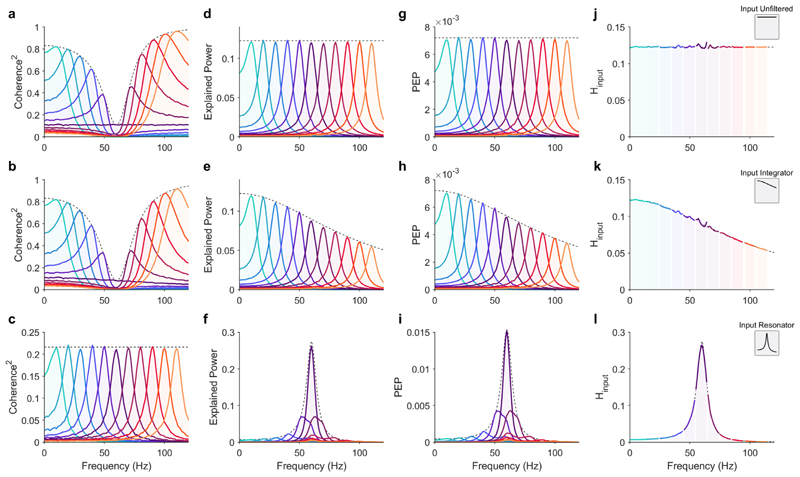
The Explained Power metric for quantifying inter-areal interactions. These simulations were identical to those shown in Figs. 1 and 2 except without 1/*f* background fluctuations. (a–c) Coherence, (d–f) Explained Power, (g–i) Proportion of Explained Power (PEP), and (j–l) estimate Input Transfer Function for 3 different input transfer functions (flat, integrator, and resonator in rows top, middle, and bottom, respectively). The flat transfer function was an all-pass filter (i.e., *H*_*input*_ = 1 for all frequencies). The integrator receiver was modeled as an exponential moving average low-pass filter (cutoff: 100 Hz), and the resonant receiver was modeled with an AR(2) model (peak frequency at 60 Hz). The dashed black line in each plot indicates the analytical result for a white-noise process with power at all frequencies equal to the max in the senders.

These results reveal the limitations of coherence to characterize inter-areal interactions when there are frequency shifts between the senders and the receiver. In our simulations, the signals the senders projected to the receiver were equal in magnitude (i.e., power at the resonant frequency), but varied in frequency. Yet, the magnitude of coherence depended systematically on the frequency of the sender (Fig. 5(a)–(c)).

Magnitude-squared coherence is a meaningful measure in the sense that it quantifies the proportion of explained variance at each frequency. However, coherence may not be physiologically meaningful even when it is close to one. For example, the sender may explain all of the power in the receiver over a particular frequency range, but those frequencies may only account for an arbitrarily small fraction of the total power in the receiver.

Considering that neurons in the receiver will be driven (at least to some extent) by membrane potential fluctuations at all frequencies, it is reasonable that the physiological impact of the sender on the receiver depends on the extent to which the sender changes the receiver’s power spectrum. This motivates an alternative measure of inter-areal interactions, which we refer to as Explained Power (Fig. 5(d)–(f)). Explained Power *E*_12_ is computed by taking the product of coherence $C_{12}^{2}$ interpretation of the references to color in this figure legend and the receiver’s power spectrum *S*_22_,

(51) $$E_{12}\equiv C_{12}^{2}S_{22}.$$

Explained Power (EP) should be interpreted as explained variance per frequency (see Eq. (23)). However, in the absence of 1/*f* background fluctuations, EP is equivalent to the signal projected by the sender scaled by the connectivity weight *w*^2^ and the receiver’s input transfer function $H_{input\;}^{2}$,

(52) $$E_{12}=w^{2}S_{11}H_{input\;}^{2},$$

(5 g–i; see Section 2). Furthermore, normalizing EP by the sender’s power spectrum EP provides a method for estimating the receiver’s input transfer function (ITF) *H*_*input*_,

(53) $${\hat{H}}_{input\;}^{2}=\frac{E_{12}}{S_{11}}$$

(54) $$=w^{2}H_{input\;}^{2}.$$

A unit-less measure of Explained Power can be obtained by normalizing *E*_12_ by the integral of the receiver power spectrum,

(55) $${\tilde{E}}_{12}=\frac{E_{12}}{\int S_{22}(f)df}.$$

We refer to the resulting EP measure after normalization as the Proportion of Explained Power (PEP) (Fig. 5g–(i)), Note that the time-domain variance of a time-series equals the integral of its power spectrum (Eq. (23)). Thus, PEP quantifies the proportion of the total variance explained by the sender per frequency. For example, a PEP equal to 0.01 indicates that the sender explains 1% of the receiver’s total signal energy at that frequency. However, in some cases, changes in PEP may be difficult to interpret. For example, such a situation may arise when the integral of the receiver’s PSD (i.e., variance) changes due to a change in the power of a single frequency band. Therefore, EP, rather than PEP, is preferred when comparing two conditions with unequal (total) variance. PEP has a straightforward relationship to an acausal linear prediction kernel where the integral of PEP equals the total explained variance (see Section 2.4).

To illustrate the utility of EP, PEP, and ITF compared to coherence, we repeated the bivariate sender-receiver simulations varying the sender frequency for three kinds of receivers: integrator, resonator, and frequency invariant (i.e., a flat input transfer function). Note, these simulations were identical to those shown in Figs. 1 and 2 except without background 1/*f* fluctuations.

In the case of the integrator receiver, coherence increased with a greater frequency mismatch between the sender and the receiver (Fig. 5(a)). By contrast, EP scaled according to the integrator receiver’s input transfer function, which in this case, was a low-pass filter (Fig. 5(d)). In the case of a resonant receiver, coherence was invariant to the sender oscillation frequency, but again, EP followed the receiver’s input transfer function (Fig. 5).

Lastly, we compared the integrator and resonator input transfer functions to a receiver with a flat transfer function (equivalent to an all-pass filter). In the case of a flat input transfer function coherence increased similarly to the integrator receiver (Fig. 5(c)). Accordingly, EP was invariant to the oscillation frequency, that is, the receiver’s transfer function did not distinguish between frequencies of the senders (Fig. 5(f)).

These results demonstrate that in all cases, EP follows the receiver’s input transfer function (Fig. 5(d)–(f)), whereas coherence predominantly depends on the receiver’s power spectrum and how the receiver’s input transfer function alters the power of the input (Fig. 5(a)–(c)). Using EP, the input transfer function could, for all three cases, be reliably estimated (Fig. 5(g)–(i)). For empirical data, the ability to estimate the input transfer function will depend on a high signal-to-noise ratio, which is not always given in real data (for further caveats on how to compute EP, PEP, and ITF in practice, see Section 2.4). Thus, in a typical experiment estimating the ITF may only be possible in a relatively narrow frequency range when the sender has the most power. However, we show that within this narrow frequency range around the peak frequency of the sender, the ITF can be reliably estimated (Fig. 5(j)–(l)). Therefore, estimating the entire bandwidth of the ITF will depend on the experimenter’s ability to evoke oscillations across the frequency range of interest.

## 4 Discussion

### 4.1 Summary

Coherence and Geweke–Granger-causality (GCC) are commonly used methods to investigate inter-areal interactions (Pesaran et al., 2018). It has been proposed that inter-areal coherence reflects the flexible gating of neuronal communication (Bressler and Kelso, 2001; Fries, 2005; Varela et al., 2001). However, recent work has raised concerns regarding the physiological and functional interpretation of inter-areal coherence given the non-local nature of the LFP, referred to as Source Mixing (Buzsáki and Schomburg, 2015; Pesaran et al., 2018; Schneider et al., 2021; Schomburg et al., 2014). The Source Mixing model is based on the understanding that the LFP is the superposition of all extracellular currents in the brain (Buzsáki et al., 2012), and in particular synaptic inputs from local and remote sources (Buzsáki and Schomburg, 2015; Pesaran et al., 2018; Schneider et al., 2021). Source Mixing refers to the phenomenon whereby spiking activity in one area gives rise to correlated synaptic currents both locally and in remote areas according to that area’s pattern of anatomical connectivity. Therefore, the only assumption of the Source Mixing Model is that the intrinsic activity of the sender is superimposed on the receiver.

Thus, according to the Source Mixing model, coherence is explained by linear signal mixing, and its magnitude depends on the power ratio between the sender and receiver (see Eq. (7)) (Schneider et al., 2021). Yet, several studies have observed changes in inter-areal coherence (between V1 and V4) without a difference in the peak power of V1 gamma oscillations (Bosman et al., 2012; Ferro et al., 2021). These studies did however observe a shift in the peak frequency of V1 gamma oscillations. Bosman et al. (2012) and Fries (2015) postulated that these frequency differences may in fact be the mechanism that facilitates inter-areal communication, and increases inter-areal coherence.

In this study, we investigated whether the Source Mixing model could also explain changes in inter-areal coherence with shifts in the sender frequency. As previously noted, the only assumption of the Source Mixing Model is that the intrinsic activity of the sender is superimposed on the receiver. This means coherence can be described in terms of the ratio of the observed power spectral densities of the sender and receiver, and thus does not strictly depend on the particular model that generated the spectra.

To that end, we modeled the intrinsic activity of each area using pseudo-periodic AR(2) models, which are linear time-invariant filters (LTI). We chose pseudo-periodic AR(2) models because they have been shown to reproduce the statistical properties of stationary gamma oscillations in primary visual cortex (Burns et al., 2011), and provide mean-field approximations of E-I circuits driven by stochastic input (Spyropoulos et al., 2022). Furthermore, we have previously shown that both AR(2) models and the (non-linear) stochastic Wilson-Cowan model (Wallace et al., 2011) predict similar changes in coherence as a result of a power change in either the sender or receiver (Schneider et al., 2021). Another advantage of using LTI models is that there are no cross-frequency interactions that can occur in non-linear systems, and these models provided a means to easily explore the influence of difference receiver input transfer functions on coherence.

We simulated two classes of receivers: an integrator and a resonator receiver (also modeled as LTI filters). We found that the magnitude of coherence systematically varied with the sender frequency for both types of receivers, however, the effect was in the opposite direction.

Our simulations show the integrator receiver predicts an increase in coherence with an increasing frequency mismatch between the sender and receiver, whereas a resonant receiver predicts a decrease. Therefore, the empirically observed changes in coherence with V1 gamma peak frequency shifts can be explained by the Source Mixing model with an integrator receiver (Bosman et al., 2012; Ferro et al., 2021).

Furthermore, our simulations show that coherence is not a veridical measure of inter-areal communication. The magnitude of inter-areal coherence is highly dependent on the shape of the receiver’s power spectrum, background 1/*f* fluctuations, and the receiver’s input transfer function. our results underscore the difficulty in interpreting the magnitude of coherence differences when there are frequency differences. We were motivated to develop a new measure of inter-areal interactions, which we refer to as Explained Power. We show that Explained Power maps directly to the signal transmitted by the sender filtered by the receiver. In addition, Explained Power provides a means to determine the receiver’s transfer function.

### 4.2 Resonance vs. integration

We compared the Source Mixing model with an integrator and resonant receiver. Both integration and resonance have been observed experimentally and are emergent properties of biophysical models of neurons (Hodgkin and Huxley, 1952; Hutcheon and Yarom, 2000; Izhikevich, 2001). Importantly, they represent distinct forms of neuronal excitability according to the temporal structure of synaptic input. Broadly speaking, integrators are more effectively driven by high-frequency input, and the higher the frequency the more effective the input (Izhikevich, 2001). Whereas, resonators are more effectively driven by inputs that match the receiver’s resonant frequency.

Indeed, it has been proposed that the resonant behavior of neurons may facilitate selective communication (“selective communication via resonance”) (Izhikevich et al., 2003), (see also Niebur et al., 1993). However, our simulations indicate that the increase in V1–V4 coherence with attention is not explained by selective communication via resonance (Bosman et al., 2012; Ferro et al., 2021). Rather, our results suggest V4 acts more like an integrator receiver, and this explains why coherence increases with a greater frequency mismatch between V1 and V4 gamma oscillations.

The peak frequency of gamma oscillations in V1 is also low-level stimulus features such as size, contrast, and color (Das and Ray, 2018; Friedman-Hill et al., 2000; Gieselmann and Thiele, 2008; Gray and Prisco, 1997; Henrie and Shapley, 2005; Jia et al., 2013; Murty et al., 2018; Peter et al., 2019; Ray and Maunsell, 2010; Shirhatti and Ray, 2018). It is tempting to speculate that shifting the peak frequency of the sender may be a common mechanism by which both attended and salient stimuli (top-down vs. bottom-up attention) gain a competitive advantage at the receiver. However, changes in frequency are not independent of changes in firing rate. Several studies have reported V1 firing rates increase with attention (Ferro et al., 2021; Kerkoerle et al., 2014; Motter, 1993; Roelfsema et al., 1998). This raises the question of whether the effectiveness of the higher frequency sender is not simply explained by a relative increase in the number of spikes. Taken together, the results presented here suggest the higher frequency sender is more effective at the receiver, because of an increase in firing rate or because integrators are more effectively driven by higher frequency input.

### 4.3 Coherence and its utility for investigating inter-areal interactions

We have shown that changes in inter-areal coherence can result from a shift in the frequency of the sender.

This result implies coherence differences do not necessitate oscillatory coupling, nor a change in the connectivity weight between the sender and receiver. Our results show that coherence is, in general, a misleading measure of inter-areal interactions, and there are numerous limitations and caveats to usefulness therein:

(1) Coherence is a strictly linear measure that captures the correlation between two signals per frequency (i.e., coherence is blind to cross-frequency interactions), thus we can expect that coherence will be most sensitive, and useful, for quantifying the linear component of inter-areal interactions. However, the conversion of sub-threshold synaptic inputs to supra-threshold output (i.e., membrane potential fluctuations to spiking) is necessarily non-linear. Thus, coherence is expected to be dominated by Source Mixing: sub-threshold membrane potential fluctuations, which do not necessarily map to, nor require, feedforward entrainment of the receiver’s spiking activity (see also Schneider et al., 2021).
(2) The usefulness of a measure for understanding a biophysical phenomenon follows from the physiological relevance of its quantification. Magnitude-squared coherence reflects the proportion of variance in the receiver explained by the sender per frequency. However, coherence is unitless, and as a result, its physiological relevance is not entirely straightforward. In fact, there is no consensus on what level of coherence is physiologically meaningful or not.
(3) Our simulations comparing an integrator and resonator receiver demonstrate that changes in coherence not only depend on the power spectrum of the receiver but also on how the receiver filters its inputs (i.e., the input transfer function). Our simulations here suggest an integrator input transfer function with a relatively weak low-pass filter (first-order, cutoffaround 100 Hz) explains the results of empirical studies (Bosman et al., 2012; Ferro et al., 2021).

However, it is worth noting that an input transfer function with a much lower cutofffrequency (i.e., an integrator with a longer time constant), would show a pattern of coherence changes similar to a resonator - at least for senders at higher frequencies. This also means that changes in power that co-occur with shifts in the sender’s peak frequency may exploit, or compensate, for the effect of the receiver’s input transfer function. Thus, interpreting coherence changes necessarily depends on knowing, or assuming, the receiver’s input transfer function.

Recognizing these limitations of coherence we were motivated to develop a new measure to characterize inter-areal interactions. We argue that the physiological significance of coherence is more easily understood in context Explained Power, or the Proportion of Explained Power at each frequency relative to the total power of the signal (PEP). Furthermore, we present a method by which the receiver’s input transfer function can be characterized.

### 4.4 Limitations of this study

This study was designed to investigate how shifts in the peak frequency of the sender may lead to changes in inter-areal coherence. We did not investigate the mechanism behind changes in the peak frequency, and therefore, our model cannot speak to the underlying mechanism of changes in peak frequency.

Our model explains an empirical observation that has been influential in the field, namely an increase in inter-areal coherence with attention (Bosman et al., 2012). It has been previously demonstrated that both coherence and Granger-causality are sensitive to changes in the power-ratio between the sender and receiver (Schneider et al., 2021). As noted however, Bosman et al. (2012) reported a change in inter-areal coherence in the absence of a change in power. The central point of the present study is that a shift in the peak frequency of either the sender or the receiver, such that the difference between their peak frequencies has changed, will influence coherence because this implies that the per frequency power-ratio between the sender and receiver has also changed. Thus, an increase in coherence and Granger-causality can occur because of a shift in the peak frequency, and in the absence of a change in the underlying inter-areal interaction. Nevertheless, our model cannot speak to the true mechanism behind changes in peak frequency nor how frequency shifts may influence inter-areal communication. Indeed, the role of frequency shifts in inter-areal communication remains an open question. Rather, our results speak to the measures that have been commonly employed in the field, namely coherence and Granger causality. Therefore, our findings show yet another important caveat to the interpretation of LFP-LFP coherence and Granger-causality (Pesaran et al., 2018).

Although we based our model on empirical studies and known biological phenomena, our model, like all models, is an oversimplification of a complex biological system. Thus, further experiments are necessary to test the extent to which frequency shifts can account for the increase in coherence with attention. For instance, one possible experiment would be to causally control the frequency of V1 gamma oscillations. This could be achieved by changing the stimulus properties (e.g., stimulus contrast) or by optogenetic manipulation. It is also worth noting that Bosman et al. (2012) only recorded the LFP, but future studies should include single-unit analyses because what matters for inter-areal communication is not coherence per se, but how neurons respond to their inputs. For example, a recent study by Spyropoulos et al. (2023) suggests that excitatory neurons in area V4 are not driven by the afferent V1 gamma oscillations, which was the case whether the monkey was attending to the stimulus or not. Even though our model may be over-simplified, it makes testable predictions and therefore may be useful to guide and interpret future experiments.

### 4.5 Outlook

Looking forward, future studies can utilize the methods presented here to more fully characterize inter-areal interactions through transfer functions and Explained Power. For instance, it may be possible to characterize input transfer functions by presenting stimuli that systematically shift the peak frequency of stimulus-driven oscillations in a sender area (e.g., V1). In practice, however, it may be difficult to simultaneously control the power of the stimulus-driven oscillations while systematically varying the frequency (Gieselmann and Thiele, 2008; Jia et al., 2013; Ray and Maunsell, 2010). An alternative approach would be to use causal techniques. This can be done at the level of sensory inputs, e.g., by stimulating the auditory or visual system with different frequencies (Arnal et al., 2019; Assaneo and Poeppel, 2018; Doelling et al., 2019; Duecker et al., 2021). The results from these studies in the visual system are in principle compatible with our source mixing model with an integrator receiver, as externally imposed gamma flicker does not interact with locally induced gamma oscillations in area V1 (Duecker et al., 2021). Another approach is optogenetics, which allows the experimenter to directly control the power and frequency of oscillations as well as the spiking activity in the sender (Cardin et al., 2009; Lewis et al., 2021). This alternative approach offers the added possibility to selectively manipulate both sub-classes of neurons (e.g., excitatory, inhibitory, or a sub-population thereof), as well as specifically target feedforward projecting neurons (Siu et al., 2021). Such experiments will be crucial to further our understanding of inter-areal communication and test the conclusions reached here, namely that increases in coherence with frequency shifts are consistent with an integrator receiver.

## Data Availability

All code was written in Matlab and run with Matlab 2021a, The MathWorks, Inc., Natick, Massachusetts, United States. The code for the numerical simulations and methods was written by the corresponding author (J.R.D) and is available upon request.
